# ESSKA consensus initiative: why, when and how?

**DOI:** 10.1186/s40634-023-00664-2

**Published:** 2023-10-06

**Authors:** Philippe Beaufils, David Dejour, Giuseppe Filardo, Joan Carles Monllau, Jacques Menetrey, Romain Seil, Roland Becker

**Affiliations:** 1 ESSKA Consensus Projects Advisor, 11 Rue Jacques Boyceau, 78000 Versailles, France; 2 Lyon-Ortho-Clinic Clinique Sauvegarde Ramsay Santé, 29 Avenue Des Sources, 69009 Lyon, France; 3 Applied and Translational Research (ATR) Center, IRCCS Istituto Ortopedico Rizzoli, Bologna, Italy; 4 Department of Orthopedics and Traumatology, Hospital del Mar, Barcelona, Spain; 5 ICATKnee at ICATME, Hospital Universitari Dexeus, Barcelona, Spain; 6 Universitat Autònoma de Barcelona (UAB), Barcelona, Spain; 7 Centre de Médecine du Sport et de L’Exercice - Swiss Olympic Medical Center, Hirslanden Clinique La Colline, Geneva, Switzerland; 8 Orthopaedic Surgery Service, University Hospital of Geneva, Geneva, Switzerland; 9 Division of Neurosciences and Musculoskeletal Diseases, Centre Hospitalier Luxembourg, Luxembourg City, Luxembourg; 10 Department of Orthopedics and Traumatology, University of Brandenburg, Brandenburg an der Havel, Germany

**Keywords:** Consensus, Guidelines, Delphi method, Formal Consensus, RAND/UCLA Appropriateness method

## Abstract

The goal of a Consensus in clinical practice is to provide daily practitioners with evidence- based recommendations on data from the literature, clinical expertise and expectations of professionals and patients. In this context, a consensus aligns with the principles of evidence-based medicine in clinical practice and is consequently regarded as a scientific work of a certain level of evidence (LOE). It is expected that such a project may contribute to filling the gap observed between scientific evidence and reality of the daily practice.

A Clinical Consensus is particularly needed for those topics that are of interest to daily practice but controversial due to lack of evidence, and for which expert agreement can provide valuable support in reaching conclusions.

A Consensus requires a strict methodology, based on two principles: an iterative process with independence of the involved groups and pluralism (geographical and professional representation). These processes guarantee the scientific quality of the recommendations.

Among the various consensus modalities, ESSKA has adopted the Formal Consensus derived from the Delphi method, and the RAND/UCLA appropriateness method. These two methods are complementary. The first one, based on questions-answers sets, is particularly suitable for questions of terminology, diagnosis, planning, strategy. The second one is based on the concept of scenarios, particularly adapted to treatment indications. These two methods can also be used within the same consensus.

The aim of this article is to define what is a consensus initiative, to detail the methodology ESSKA has chosen, and to point out the key role of the dissemination.

## Why should a consensus be proposed

Since the 1990s clinical research has been based on the concept of Evidence Based Medicine (EBM) [11, 19], aiming to establish scientific “certainties” from studies with a strong methodology and thus transmit recommendations that can be used by all in a broader healthcare community. As Sackett et al. [19] stated, EBM integrates *“individual clinical expertise with the best available external clinical evidence from “systematic research”* and the *“compassionate use of individual patients’ predicaments, rights and preferences. By individual clinical expertise, we mean the proficiency and judgment that individual clinicians acquire through clinical experience and practice.*”. EBM is thus a complex concept that is often wrongly perceived as exclusive to scientific studies, focusing solely on the level of evidence for each study. In particular academic research is often performed in a manner which does not reflect the daily clinical practice but rather an ideal in order to standardise inclusion criteria as much as possible and is, therefore, not EBM. Academic studies are indeed a part of EBM, which is why they are referred to as ‘scientific evidence’ rather than ‘clinical evidence,’ as argued here.

It is mainly in the field of medicine (e.g. oncology, cardiology, …) where high-level scientific evidence studies, especially blinded randomised trials, are performed. It is much more difficult in Orthopaedics and Sports Medicine to design proper randomised studies which provide robust answers for our daily practice. Judgment criteria are often functional and subjective, therefore difficult to define with precision, despite the common use of patient reported outcome measures (PROMs) which attempt to objectively quantify these criteria. A surgical intervention is a package of care that starts at the referral and finishes when the patient is discharged. It therefore has many variables. The patient, the surgical team and hospital unit contribute also to the variability [18].

Moreover, translating scientific evidence into surgical practice is a long process [10] which explains the frequent existence of a significant gap between scientific evidence and the reality of daily practice [18, 20]. Many reasons can explain this distortion: the surgeons’ practices and preferences, the societal pressure, the medico-economic healthcare system specific to each country, … and probably a certain distrust with regards to the data of science.

But it is essential that the daily actions are based on solid reasoning shared by the community, both surgeons and scientists. This is the goal of a Consensus, whose aim is to produce good practice recommendations. Good practice recommendations are defined in the healthcare sector as “*methodically developed proposals to assist the practitioner and the patient to find the most appropriate care in given clinical circumstances*” [12].

The production of a consensus is based on a strong demand from public authorities or the public or private healthcare systems around the concept of proper decision-making in choice of care with its consequences in terms of public health and health economics. A consensus can therefore be produced not only by a public health organisation, but also by a scientific society. Scientific societies are becoming more professional. Their role now goes far beyond the transmission of knowledge through academic publications or oral presentations; the production of consensus proceeds from this extension of the scientific societies’ role. In this light, the European Society for Sports Traumatology, Knee Surgery and Arthroscopy (ESSKA) decided in 2014 to promote such projects, following a specific methodology, named formal consensus [20]. ESSKA has aimed to give its members and others objective recommendations which are easy to understand and of help for surgeons to treat their patients better in “real life”.

Five ESSKA Formal Consensus documents have already been delivered (Table 1).

**Table 1:** The five already delivered ESSKA consensuses http://www.esska.org/page/projects

*Year*	*Title*	*KSSTA publication*
2016	Surgical management of degenerative meniscus lesions	Published [2]
2018	Management of traumatic meniscus tears	Published [17]
2022	Management of anterior cruciate ligament revision in adults	Published [21, 22] One other Submitted
2022	The use of injectable Orthobiologics for knee osteoarthritis: a formal ESSKA consensus. Part 1—Blood-derived Products (PRP	Submitted
2022	Osteotomy around the painful degenerative varus knee	Submitted

The aim of this article is to define what a consensus initiative is, to detail the methodology ESSKA has chosen, and to point out the key role of the dissemination.

## What a consensus is and what it is not!

### What a consensus is not

A consensus is not a systematic review of the literature, nor a meta-analysis.

Systematic reviews or meta-analyses aim to aggregate the current content of the scientific knowledge on a given subject. They follow strict rules which provide a high level of scientific evidence. This kind of academic studies are extremely important.

However, some systematic reviews or meta-analyses are simply unavailable or not feasible due to the lack of primary data. Moreover, even if the findings from these studies may be strongly positive, two points may limit their scope and may explain why their conclusions are frequently not immediately adopted in the general community:

- Selection bias of the population studied may weaken the external validity of the conclusions [20]
- Important questions for daily practice may not have been covered by the scientific literature.

### What is a consensus?

Coming back to the definition of evidence-based medicine, the consensus is the appropriate process to integrate the three pillars: “science”, “clinical expertise” and “patients’ expectations”. The target population of such an initiative is represented by the various stakeholders in the healthcare system (professionals, patients, decision-makers).

“The Consensus methods are defined as a way to synthesize information and compare contradictory opinions, with the aim of defining the degree of agreement within a group of selected individuals.”[12]

This alchemy is essential to embed the target population in the conclusions of the Consensus. However, in this process the large variety of cultural aspects linked to the geography, history, medical, and economic system of each country should be taken into account. When a Consensus is set up at a continental level like Europe, this point is of paramount importance. A surgeon from northern Europe does not necessarily address concerns in the same way as a surgeon from southern Europe. The truth is not on one side or the other and an important process of mutual understanding and agreement is essential to achieve the project.

Moreover, a consensus must follow precise methodological rules to guarantee the greatest possible “objectivity”. Introducing expert opinion in the process of scientific production may be seen as a weakness. However, consensus is definitely not a meeting of experts around a table on a given day, who produce recommendations (that would be simply an expert opinion). To reach the status of a Consensus, a strong methodology must be applied which addresses this criticism and diminishes the biases as much as possible. The key factors are such as pluralism of the involved people with the concept of agreement around contradictory opinions, iterative processes involving independent groups and representativeness. The independence between the different groups avoids a single group being judge and jury.

In 2020, six years after the ESSKA formal consensus launch and in order to address the scientific cultural link and the methodology, ESSKA decided to create a position of ESSKA consensus projects advisor whose role was to standardise the methodology among the different consensuses, assist the chairpersons in organisation and methodological processes and facilitate the link with the ESSKA board. This person is of course not involved in the scientific content and may be thus seen as a neutral contributor who guarantees as much as possible the “objectivity” of the process, regardless of the topic.

**Fig. 1:**
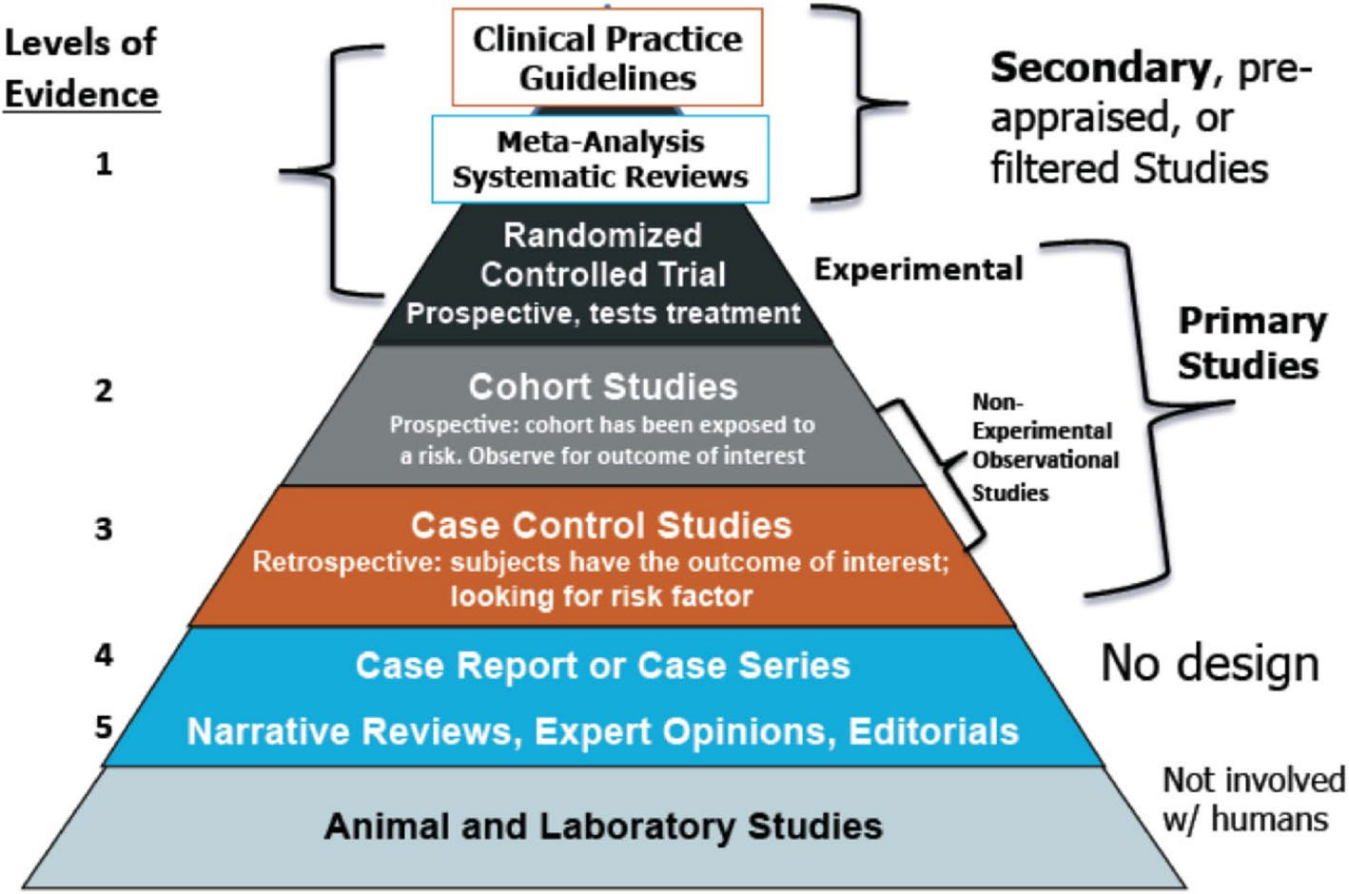
Evidence based medicine pyramid showing clinical practice guidelines as level 1. Reprint from Forrest J and Miller S [9]

Following this methodology, a Consensus can be definitely considered as a level 1 scientific work [9] (Fig. 1).

But consensus is not an imposed truth. In no case does it imposes itself neither in space outside its defined geographical framework, nor in time (recommendations of one day may be superseded in the future).

## When is a consensus recommended ?

Not all topics are suitable for a consensus.

A subject which already has a large, well documented, high scientific evidence does not need a supplementary consensus. Moreover consensuses are not intended to describe the entire management of a health condition or disease. They should be limited to controversial points for improvement in management, identified using studies of clinical practice or, in the absence of such studies, using the opinions and experience of healthcare experts in the subject. Topics such as management of First Patello Femoral Dislocation, or First Anterior Shoulder Dislocation or Groin Pain, which will be finalised soon, do match these criteria.

The choice of the subject and the exact definition of the framework are thus a very important preliminary step. This is achieved within ESSKA by asking the sections, committees, or working groups to make proposals. They are then discussed among the ESSKA board members and a final list, taking into account the pertinence of the subject and the feasibility, is finally decided on the strength of the proposal.

## The methodology of a consensus

### General principles

A consensus requires.

- Simple, practical questions, corresponding to everyday expectations
- A rigorous methodology based on iteration and on the concept of agreement
- A rigorous literature review
- A geographical representativeness at all levels of the process. The geographic representation allows the cultural mix mentioned above. Moreover, it facilitates the dissemination of information among the different countries at the end of the process. ESSKA decided to involve in the process all the affiliated national societies
- A professional representation: different specialties depending on the subject, different scientific levels from the “scientist” to the “daily practitioner” must be involved.

### The main consensus models

The consensus method is usually based on an iterative process which allows for several rounds within different independent groups of experts to gradually reach an agreement and propose recommendations. Four consensus methods are traditionally described [3, 16]: Delphi [4], nominal group [5], RAND / UCLA Appropriateness method [8], consensus conference [15].

The Delphi method is the most frequently used. Its name is derived from the oracle of Delphi known for her prophecies delivered under divine possession by Apollo. As already above mentioned, the method assumes that group judgements are more valid than individual judgements. Experts answer questions in two or more rounds. After each round, a facilitator summarises answers and reasoning from previous round. Experts are then asked to revise earlier answers in light of replies of other experts, until the answers converge and consensus is reached. But this theoretically valuable method has not always been undertaken properly and has intrinsic limitations, when used at an international level, of disagreement or misunderstanding due to inconsistent terminology; the “Babel” syndrome of intercultural and multilingual collaboration.

The following paragraphs describe the ESSKA Formal Consensus process [13] derived from the Delphi Method and incorporating a stronger methodology, and the RAND / UCLA method [8], based on the scenarios appropriateness. Both these methods have been adopted by ESSKA.

### The ESSKA formal consensus method

The Formal Consensus method was proposed by the High Authority of Health (HAS – *Haute Autorité de Santé*) in France. It is derived from the Delphi method and the following comments are directly inspired by documents produced by HAS [12, 13]. Its purpose is to formalise the degree of agreement among experts by identifying and selecting, through iterative ratings with feedback, the points on which experts agree and the points on which they disagree or are undecided. The guidelines are subsequently based on agreement points.

The recommendations must be concise, based on the formal agreement of experts and, according to the literature available, with the levels of evidence identified, unambiguous, and clearly respond to the questions raised.

#### The groups

Chairpersons, Steering groups, Rating Groups, and Peer Review groups are all involved in the process. The two chairpersons define the subject in conjunction with the ESSKA board. The consensus advisor checks the feasibility and defines with the chairpersons the framework of the Consensus (inclusion and exclusion criteria). The chairpersons propose the list of the steering group, the rating group and the peer review group in conjunction with ESSKA.

##### Steering group (10 to 12 persons)

The Steering Group includes professionals having expertise in the field and the skills to manage a group with potential diverging interests. It is initially divided into two subgroups, the Question Group, and the Literature Group which should work independently at the beginning of the process (see below). Authority, impartiality, moderation, and ability to synthesize are the required qualities.

The missions of the steering group are to:

- Propose a list of questions
- Perform a critical analysis of the available literature
- Propose question–answer sets (recommendations or statements) to the rating group
- Organise the rating rounds among the rating group
- Draft the version of recommendations submitted to the peer review group
- Finalise the text

##### Rating group (20 persons)

The rating group is made of persons who are directly involved with the clinical subject in their daily practice. The group should be multidisciplinary when needed and geographically well distributed. Members of the rating group cannot be included in the Steering Group or the Peer Review Group, to guarantee the complete independence of these groups.

The mission is to review the first draft in a two-round process.

##### Peer review group (50 persons)

The peer review group gives a formal opinion on the content and form of the version after the rating rounds, in particular its geographical applicability, and readability. Members of this group cannot be members of the Steering or Rating Groups. Involving representatives of ESSKA’s affiliated national societies is the proper way of setting up this group and facilitates further dissemination of the consensus.

#### The process

**Fig. 2:**
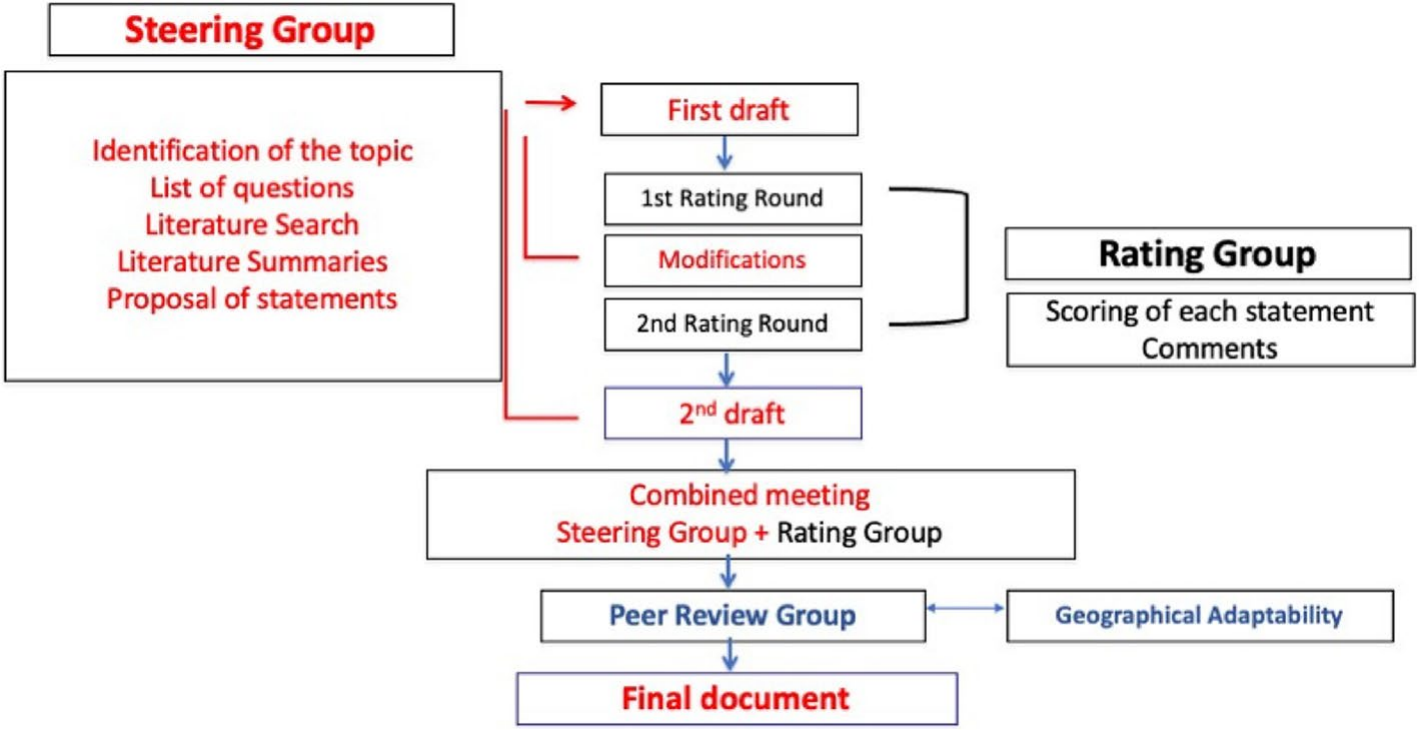
Algorithm of the ESSKA formal consensus process

There are five phases (Fig. 2).

##### Phase 1. Question list and literature search (Steering group)

This phase is undertaken by the Steering Group following the input of the Question and Literature Review Groups.

The list of questions proposed by the question group must correspond to the expectations of the daily practitioners and not necessarily to the content of the literature. This is followed by the literature search done by the Literature Group which establishes the search strategy and produces literature summaries according to the list of questions. Statements for each question are then proposed by the question group and validated by the whole steering group. For each question–answer set, a grade of recommendation is provided:

- Grade A: high level of scientific support
- Grade B: scientific presumption
- Grade C: low level of scientific support
- Grade D: expert opinion

##### Phase 2. Rating phase (Rating Group) [14]

This phase is carried out by the rating group. In this phase, the statements on which members of the rating group agree and those on which they disagree or are undecided, are identified by means of a score conducted in two rounds with an interim feedback meeting. For each question–answer set, it is possible to assign a score from 1 to 9 together with a box for comments by the raters:

- Totally inappropriate (or unacceptable) …0.1
- Intermediate levels ………………………2–8
- Totally appropriate………………………..9

This process is conducted in two rounds. In the first round, each proposed statement is valued by summing the rater values and defined as appropriate, uncertain, or inappropriate by the following criteria:

- Appropriate: median value ≥ 7 and the scores are all ≥ 5
- Uncertain: median value 4 to 6.5
- Inappropriate: median value ≤ 3.5 and the scores are all ≤ 5

An intermediate meeting of the steering group can decide to include or not the comments of the raters in the statements, especially those which did not reach a strong agreement. Statements for which there is a strong agreement are accepted as they are.

In the second round, the remaining modified question–answer sets are discussed according to the same rules. A final combined steering-rating groups meeting is organised in order to reach a final consensus.

##### Phase 3. Drafting the final consensus document

The chairpersons of the Steering Group draft the final consensus document to be validated by the Steering Group and submitted to the Peer Review Group.

This draft should include:

- An introduction defining the topic with inclusion and exclusion criteria, reminding the current knowledge and/or lack of knowledge, providing some definitions if necessary, and providing the methodology with the list of names of the different groups
- A list of question–answer sets. Each question–answer set is written as follows:
  - Question
  - Statement
  - Grade of recommendation
  - Literature summary with the level of scientific evidence
  - Reference list

##### Phase 4. Peer review

The document is sent to the members of this group for checking geographical adaptability and readability.

##### Phase 5. Finalisation

The final version (long text with all the questions-answers, literature summaries and reference list) and a summary of the consensus are drawn up. The validated versions of these two documents are disseminated.

### RAND/UCLA appropriateness method

The ‘RAND/UCLA Appropriateness Method” (RAM) was developed by RAND and UCLA in the 1980s [8]. It has been further developed and refined in North America and in Europe. Several RAND-UCLA consensuses in the field of musculoskeletal diseases have already been published [1, 7, 22]. The aim of this type of consensus is to develop patient-specific recommendations about the appropriateness of a specific procedure for different clinical scenarios by combining the best available scientific evidence with the collective judgement of a panel of experts.

#### Basic steps

**Fig. 3:**
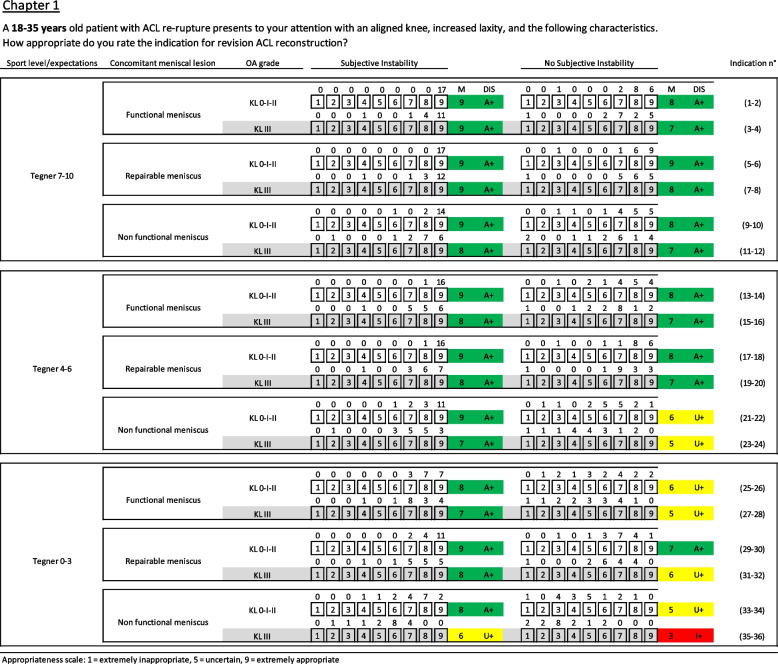
Example of Rand scenarios (ACL Revision ESSKA consensus). Clinical scenarios for the age range 18–35. Reprint from Management of anterior cruciate ligament revision in adults: the 2022 ESSKA consensus part III-indications for different clinical scenarios using the RAND/UCLA appropriateness method [22]. M: Median value, DIS: Disagreement, A: Appropriate, U: Uncertain, I: Inappropriate, + : Without disagreement, -: With disagreement, Green: Appropriate scenarios, Yellow: Uncertain scenarios, Red: inappropriate scenarios

A detailed literature review is performed by a core panel to synthesise the best available scientific evidence on the procedure to be rated (for example cartilage treatment [7], meniscal lesion treatment [1], ACL revision [22], and PRP injections). At the same time, the core panel produces a list of specific clinical scenarios or “indications” in the form of a matrix which categorises patients who might apply for the procedure in question in terms of their characteristics e.g. symptoms, past medical history, results of relevant diagnostic tests, specific pathological features, and expectations. These indications are grouped into “chapters” based on the primary presenting aspect characterising the patient being referred for treatment or considered for a particular procedure (Fig. 3).

The Rating Group comprising a panel of experts is identified. The literature review and the list of indications, together with a list of definitions for all terms used in the indications list, are sent to the members of this panel. For each indication, the panel members rate the appropriateness of the procedure on a scale of 1 to 9, where 1 means that the expected harms greatly outweigh the expected benefits, and 9 means that the expected benefits greatly outweigh the expected harms. A median rating of 5 can mean either that the harms and benefits are about equal or that the rater cannot make the judgement for the patient described in the indication.

The panelists rate each of the indications twice, in a two-round process. In the first round, the ratings are made individually at home, with no interaction among panelists. In the second round, the panel members meet for 1–2 days under the leadership of a moderator who is experienced in using the method. Each panelist receives an individualised document showing the distribution of the experts’ first round ratings; together with their own specific ratings. During the meeting panelists discuss the ratings, focusing on areas of disagreement, and are given the opportunity to modify the original indications and/or definitions, if desired. After discussing each chapter in the list of indications, they re-rate each indication individually. No attempt is made to force the panel to a consensus. Instead, the two-round process is designed to sort out whether discrepant ratings are due to real disagreement over the use of the procedure (“real” disagreement) or to fatigue or misunderstanding (“artifactual” disagreement).

**Fig. 4:**
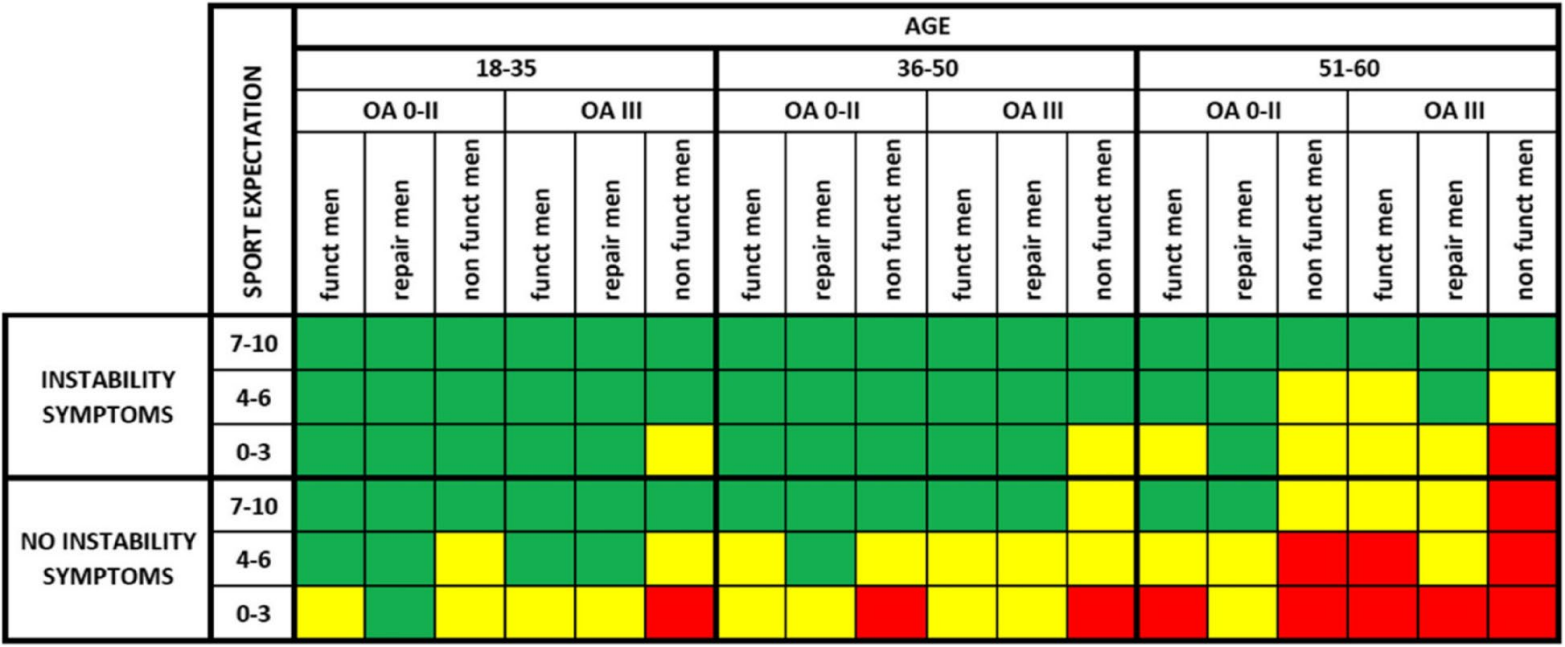
Graphic representation of the overall RAM consensus results on the appropriateness of ACLRevision in adults. Reprint from Management of anterior cruciate ligament revision in adults: the 2022 ESSKA consensus part III-indications for different clinical scenarios using the RAND/UCLA appropriateness method [22]. Green: Appropriate; Yellow: Uncertain; Red: Inappropriate, INST: instability symptoms

Finally, each indication is classified as “appropriate,” “uncertain” or “inappropriate” for the procedure under review in accordance with the panelists’ median score and the level of disagreement among the panelists (Fig. 4):

- Appropriate ……………7 to 9
- Uncertain ……………..4 to 6
- Inappropriate …………1 to 3

### RAND method versus “Formal Consensus” method

These two methods are not mutually exclusive but complementary. They are both based on a comprehensive literature review and clinical expertise. They both rely on an agreement evaluation provided by an expert panel in a two round process.

Both methods can be used in the same Consensus [1, 21, 22], RAND being preferred for the”Indications of procedures” sections and Q&A Formal Consensus Model for other sections such as terminology, diagnosis, planning, and strategy.

## Dissemination

Dissemination is an essential step. It allows practitioners and the public to adopt the recommendations generated by consensus. All possible media should be used.

### Documents

Several documents are usually produced:

- *Long consensus document.* This is reported on the Society website which should be considered as a scientific reference [6].
- *Short document*. After a short introduction explaining the objectives, it includes all the Q&As with their grade of recommendation or scenarios and their appropriateness level. The list of bibliographic references is also provided.
- *Flyers* in congresses, papers in ESSKA news.
- *Reports* at congresses and conferences of scientific societies, ESSKA of course, but also any other congress or scientific society wishing to disseminate information. It is essential that these presentations are stamped ESSKA (with copyright) and that the speakers, members of the steering group, abide by this rule.
- *Scientific publications.* ESSKA consensuses are published in ESSKA journals and must follow the guidelines of a scientific article. They follow the standard peer review process of the journal. But insofar as the recommendations of the consensus cannot be the subject of requests for modifications by the reviewers (by definition, they cannot be modified), it is very important that the editorial board is associated upstream in the consensus building process. Finally, the article(s) is (are) best published as Open Access. This promotes accessibility by readers and allows reprints in different languages ​​in other scientific journals.

### Dissemination among surgeons

ESSKA has a pre-eminent role (website, ESSKA news, ESSKA journals, ESSKA academy, congress, etc.) but national affiliated scientific societies must also be widely sought to contribute. Hence the interest in taking from the peer review group members of national affiliated societies. The information is better disseminated through them. It will be facilitated by the fact representatives of the affiliated national societies are involved in the peer review process. It is imperative that this distribution be made under ESSKA copyright conditions.

### Miscellaneous

Dissemination should be also considered in other medical specialties, other European or International Orthopaedic or Sports Medicine Societies, patients associations, public and healthcare authorities at both National and European and level.

## Conclusion

By developing the production of clinical practice consensus, ESSKA wishes to provide its members and, more generally, the orthopaedic community with clear and reasoned answers on topics that meet the concerns of daily practice in the hope that the adoption of thoughtful practices that scientific evidence alone does not always achieve. Five ESSKA Consensuses have already been produced. Five others are in the process of finalization at the time of this publication. In response to strong demand from surgeons and other healthcare professionals, from patients and healthcare authorities, as a result of this initiative, this format will develop even more in the future. It is important to consider the limitations of this exercise which represents a picture of the best possible practice recommendations at a given moment in time. These recommendations will evolve over time due to changes in response to scientific advances, policy changes, resource constraints, and innovations, regular planned reviews will be required with a revised version developed as necessary.
